# Diet of Charitable Institutions
*The Cheapest and most Nutritious Food for Charitable Institutions and the Poor. By C. H. F. Routh, M.D. pp. 60. London: 1854.


**Published:** 1856-04

**Authors:** 


### DIET OF CHARITABLE INSTITUTIONS.*

Dr. Routh, having been requested to provide a new Diet List for the inmates of the Hill Street Female Refuge, in London, has not only done so, but has published, in a very readable form, the results of the inquiries which he was led to make into the alimentary value of various articles of food. He has taken into consideration: 1. The relative value of different articles of alimentation; 2. The quantity and quality of food; and 3. The diet recommended.

In speaking of tea, the author shows that there is a great waste of its nitrogenised constituents in the ordinary infusion; and he suggests the use of tea-bread—a modification, in fact, of the brick-tea which is eaten by the Mongols and other neighbouring races:—

"If the refuse of leaves left in the tea-pot be carefully collected, dried, and reduced to an impalpable powder, and mixed with flour, —and especially that of the poorer kinds of grain,—the mixture makes a highly nutritious bread. I have made the experiment with one-third tea and two-thirds flour. The result is, a very edible bread, but with a black colour and a very strong taste of tea. A smaller quantity should therefore be mixed, and the result would be, a bread having about 20 per cent. of nitrogenous matter, in lieu of only 16, which the best white bread contains."

This is an important suggestion, which might, especially in times of scarcity, be turned to useful application. To show what an amount of nutriment might be thus applied, Dr. Routh calculates that, from January 1836 to January 1850, the amount of waste in the tea imported was 45,566,119 lbs. of caseine, and 3,261,794 lbs. of theine—both nitrogenised, and hence nutritive constituents.

This is but one among many important remarks with which the work abounds.

---

* The Cheapest and most Nutritious Food for Charitable Institutions and the Poor. By C. H. F. Routh, M.D. pp. 60. London: 1854.

